# A Rat’s Progress: Plague and the “Migratory Rat” in British India, 1896-1899

**DOI:** 10.1093/jhmas/jrae044

**Published:** 2024-12-11

**Authors:** Christos Lynteris

**Affiliations:** University of St Andrews, Scotland, UK

**Keywords:** rat, colonialism, plague, India, movement, space

## Abstract

Whether referring to oceanic travel on board of ships or to movement in terra firma, framings of the “migratory rat” formed a key epidemiological component of approaches to the Third Plague Pandemic (1894-1959) as the first pandemic to be understood as caused by a zoonotic disease. In this article, I examine the emergence and development of scientific framings of the migratory rat in the first, explosive years of the third plague pandemic in India (1896-1899). Examining publications and archival sources, I ask how this animal figure came to inform and transform epidemiological reasoning. Going beyond established approaches that have shown how the rat-plague relation was mobilised by colonial doctors to pathologise Indigenous lifeways, I argue that more complex and ambivalent processes were also set in motion by this figure. First, I show how the migratory rat became invested with attributes of invasiveness that assumed ontological qualities in colonial epidemiological reasoning. Second, comparing the migratory rat with the hitherto established “staggering rat,” I argue that the former embodied new approaches to both space and time in epidemiology. Third, I show how Indigenous scientists came to mobilise this complex figure to contest colonial approaches to plague.

Ideas of species invasiveness and disease invasiveness are today closely associated with images of “exotic” animals, plants, and microbes, in other words lifeforms introduced from far away, through long-distance travel, in many cases such that could not have been achieved without human (voluntary or involuntary) assistance. As other articles in this special issue show, this image of the introduction of exotics from far-away lands, cultures, and soils is tightly connected with nineteenth-century framings of migration, colonization, climate, and race, as well as with Victorian concerns and desires regarding the mastery of nature. While aptly describing hegemonic and xenophobic framings of invasiveness, exotics-focused approaches to the history of scientific approaches to species and disease invasiveness risks overlooking other processes involved in their emergence and development: in particular, ones focused on smaller scales, local forms, and processes of animal, plant, or microbial mobility. In this article I examine the case of the “migratory rat” from this perspective and explore the ways in which it came to inform colonial ways of reasoning about the propagation of plague in India.

Today the image of the migratory rat is well-engrained in modern imaginaries, and is especially connected to configurations of the animal as a seafaring intercontinental transmitter of bubonic plague. This idea was formed during the height of the third plague pandemic (1894-1959), a global pandemic of plague that caused more than twelve million deaths. It takes the rat to be an animal whose global movement has not only led to the spread of the disease to all inhabited continents since 1894, but also to the dissemination of the second plague pandemic, and its world-catastrophic effects summarized since the mid-nineteenth century under the moniker of the Black Death.^1^ Rats’ global movement is thus generally understood in terms of a mammalian invasion that, being simultaneously a bacterial one, has posed time and again a severe threat to humanity.^2^ Historians and biologists have been recently debating the actual role of rats in the second pandemic, asking whether this may have instead spread by means of other animals, including human ectoparasites.^3^ Still, the image of the migratory rat as a destructive force of world-historical proportions remains a key component of our pandemic imaginary and of ways in which the risks involved in human-animal relations are framed and experienced within modern, technoscientific societies.^4^

Recent works have contributed new understandings of how this image of the globe-trotting, pestilential rat emerged not as a result of lab-based discoveries, but in the context of practical efforts to control and contain the march of the third plague pandemic. In particular, these studies have focused on how the rat was rendered an epidemic villain through the development and application of techniques and technologies aimed at controlling the animal, even when scientific proof of its involvement in the spread of plague remained lacking or contested.^5^ Discussing the first half of the twentieth century, my own work on the subject has focused principally on the epidemiological problematization and international control of the maritime transport of rats, which in turn became a “pandemic infrastructure” that allowed the development of new forms of human/non-human mastery.^6^

By contrast, in this article, I explore a historical process that predated the development of the idea of the rat as a maritime or indeed pandemic spreader of plague: the emergence of the migratory rat as a concern regarding the land-based spread of plague within and between locations in British India in the initial years of the third plague pandemic (1896-1899). The examination of the emergence of this terrestrial, pestigenic figure of the migratory rat allows us to see that ideas of species and disease invasiveness have not been necessarily tied to long-distance travel or framings of exotics. Indeed, they have also formed key areas of scientific concern and investment within short-distance scales and without reference to lifeforms imagined to be geographically, biologically, or symbolically, out of place.

While revealing and making a first attempt at covering the historiographical gap relating to the medicalization of local animal movements, this article studies the case of the migratory rat so as to show how, during the first years of the third plague pandemic in India, animals, disease, and space became entangled in novel ways within emerging forms of epidemiological reasoning. In terms of bringing rats, plague, and space together, I argue that the migratory rat challenged and replaced an earlier rat-figure, which had emerged two decades earlier, at the dawn of the third pandemic: the “staggering rat.”^7^ Reconfiguring the movement of rats in response to the disease, and embodying what I call a new kinetic dramaturgy of plague, the migratory rat reflected and fostered new ways of thinking about epidemic diseases, while retaining and building upon previous concepts of rats as animals that are ontologically conditioned by plague. Examining reports, newspaper articles, and the proceedings of the Indian Plague Commission, with a key focus on Bombay and its surrounding area, I argue that the new kinetic dramaturgy relied on ideas about the “Rat’s Progress,” or the continuous movement from place to place of plague-infected rats in an ill-fated attempt to escape the disease, and by so doing spreading it to new localities.

## Plague and Rats in India

Historians of plague India have over several decades examined the impact that plague had on both public health and colonial policy in the British colony, as well as Indian responses to British anti-plague measures.^8^ More recently, epistemologically-oriented attention has also been paid to the development of scientific knowledge and framings of the disease that caused over ten million deaths in the subcontinent in the course of its third pandemic.^9^ A common topos of both the traditional, socio-politically oriented, and the new, epistemologically-oriented historiography of plague in India is space, its problematization and management. In the course of the third pandemic’s impact in India, plague was seen, to borrow a term from Prashant Kidambi, as an “infection of locality;” an approach allowing what David Barnes has coined a “sanitary-bacteriological synthesis,” which fostered anti-plague measures focused on disinfection, as well as the destruction of infected houses belonging to Indigenous individuals and communities.^10^ This configuration of plague as an infection of locality brought together old ideas about miasma with new ones about bacteria.^11^ It was a synthesis particularly fostered by the Pasteurian notion, first developed during the 1894 plague outbreak in Hong Kong, that the natural reservoir of plague bacteria was the soil, where the disease could be maintained in attenuated form over long periods and from which it could recrudesce and spread back to humans as well as to animals.^12^

However, plague was also configured as a disease *between* localities, or to be more precise as a disease that connected different spaces and localities in ways that needed to be urgently rendered epidemiologically intelligible and actionable. By 1896, when plague had spread from Hong Kong to Bombay, an old question that had time and again been the subject of fierce scientific debate and political controversy resurfaced as a key to understanding and containing the disease: Who or what “propagated” (the verb used at the time) plague between locations? This simple but notoriously hard to agree-upon problem contained many more questions of increasing complexity following the identification of plague’s causative bacterium in 1894. Were the same agent or agents responsible for both the short and long distance propagation of the disease? And was it the same agent that propagated plague over land and sea? Contrary to what might be expected, it was not maritime but overland propagation that formed the main concern of medical scientists and public health officers over the first, explosive years of the third plague pandemic in India (1896-1899). While the role played by maritime travel and trade in the arrival of plague in India was discussed during that period, what formed a much more pressing question from the practical point of view of colonial administration was containing the disease: How was it spreading inland across British India, after it first arrived there in September 1896?

Though not yet considered to be a *sine qua non* of plague outbreaks, during this period rats became gradually framed as “agents of plague.”^13^ The importance placed on rats in this respect varied from mild interest in them as co-patients to strong takes on their role in human epidemics of the disease. In all cases, however, what I have elsewhere coined as the “epidemiological dividual” role of rats was maintained.^14^ Rather than being seen as the indispensable plague protagonist (a role developed after 1900), the rat was instituted “as an epidemiologically knowable and actionable propagator of plague through investigating the complex ways in which it related to other potential hosts or vectors of the disease.”^15^ It was in the relation between rats and soil, cloths, humans, and grain that the actual “agency” of plague was sought out, and where anti-plague operations tried to intervene. Thus, if in the context of the 1896-1899 epidemic of plague in India the movement of rats became a productive problem for framing the disease, this was not because they were seen as the only or main source of the bacillus, but because rats were seen as being uniquely able – as mobile “vermin” long-invested with notions of mischief, sagacity, and transgression – to connect different agents of the disease both in and between localities.^16^

Epidemiological and public health concerns over what soon came to be known as the migratory rat were focused on two parallel but interlinked questions: did rats propagate plague from house to house, and did they propagate the disease from village to village? The Report of the Bombay Plague Committee (1897-1898) aptly portrays the productive uncertainty surrounding these questions:

Whether rats bring Plague from infected into uninfected localities, or whether local rats are the first victims to existing local infection, has not been determined. It seems almost certain that, in some instances, rats suffering from Plague have moved in numbers to a fresh locality, and have brought Plague among the people there. In other instances the evidence seems to show that local rats sickened in consequence of the introduction of infection by human agency.^17^

One might expect that this idea of land-based rat migration would be linked at the time to debates developed by natural historians over the past hundred years or so concerning the supposed disappearance of black rats (*Rattus rattus*) in Europe, as a result of the invasion of the continent by brown rats (*Rattus norvegicus*) by means of what is often described as a “mass migration” across the Volga in the 1720s.^18^ Much debated by naturalists since its introduction by Peter Simon Pallas, this mass species invasion later came to be framed as being the reason for the end of the second plague pandemic in Europe.^19^ However, there is no trace of an impact of this theory or story in the plague literature in India between 1896 and 1899. Rather, the figure of the migratory rat in this case was rooted in what Nicholas Evans has identified as “the interlinking of animal and human agencies and responsibilities at a time of epidemic disease.”^20^

## “Rat’s Progress”

In one of the first authoritative accounts of the epidemic, the Municipal Commissioner of Bombay asserted that the direction of the spread of the disease “was preceded by a migration of rats from the parts of the city which were most affected.” “By the commencement of December nearly all the rats had disappeared from Màndvi and adjacent quarters of the city, while they were noticed in Kàmàtipura, Tàrdeo and Byculla in great numbers, many of them being found dead. The bubonic plague followed in their track with unerring regularity.”^21^ So struck was the commissioner by this reported phenomenon that he proceeded to make verbal inquiries among the local population. The results he acquired were said to be invariable: following the plague outbreak at the center of Bombay, rats disappeared from this location, and “were observed in great numbers on the west and north” of the city.^22^ For the commissioner, this was sufficient evidence as regards the origin of outbreaks of the disease in these areas. Here, in this short sequence of epidemiological reasoning, we find all the elements composing the migratory rat as an explanatory device for the simultaneous spread of plague from one area to many others, often at varying distances between one another, within a given location. Yet we should not be tempted to reduce the migratory rat to a figure, which, once added to the profile of a given outbreak, simply resolved the question of its temporal-spatial distribution. For at the same time as the migratory rat provided solutions in terms of epidemiological reasoning, it also produced important questions for the latter, fueling complex medical and public health debates in the colony.

One of the phenomena that perplexed those studying and managing plague in India was the alleged rapid disappearance of rats soon after the beginning of human outbreaks in any given location. This was a phenomenon that, some reasoned, could be explained as the result of rats migrating under the bane of an epizootic. In his breakthrough (although now shown to be fraudulent) article on the transmission of plague via rat fleas, published in October 1898, Paul-Louis Simond attributed the end of epidemics in a given area at least partially to the massive flight of rats supposedly following the rise of an epizootic.^23^ While this may be good news for a given location, it was bad news for others, as it was feared that rat flight resulted in the spread of the disease in the locations invaded by the escaped rodents. By the end of 1897, arguments that rats conveyed the disease from house to house and from district to district within a given village town or city drew support from evidence systematized by the Collector of Thána, A.C. Logan.^24^ Coining this process the “Rat’s Progress,” Logan relied on the observations of Surgeon-Major A.V. Anderson, Deputy Sanitary Commissioner of the Western Registration District. Anderson was a proponent of the, at the time popular, dichotomous theory of the propagation of plague: “With regard to the mode of conveyance of bubonic plague from place to place, it would appear that towns and villages remote from an infected centre become infected by human intercourse, while contiguous places frequently become infected by rats carrying and propagating the disease.”^25^ Anderson observed the plague outbreak in Bándra Municipality, a coastal suburb of Bombay, where the disease first broke out on 3 December 1896, in a house where ratfalls had been observed seven days earlier. Then the same pattern repeated itself in areas adjacent to the suburban centre, in all cases with ratfalls preceding human cases by seven to ten days. Anderson speculated that, “the Bándra rats spread the disease in the adjacent villages as they entirely disappeared from the town.”^26^ Based on this information, as well as on remarks by Anderson that rats had also deserted the Maharashtra city of Bhiwandi (also in Thána district) when this was affected by plague, Logan exercised his epidemiological imagination so as to generate a transferrable outbreak narrative. Noting that, “It is of course not likely that these sagacious animals would remain in a plague-stricken place till they were all exterminated,” he argued that rats introduced the disease through a South to North “exodus” meant to evade plague, but effectively spreading it through “successive quarters of the town, infecting one after the other.”^27^ Endorsed by the Executive Health Officer of the Bombay Municipality, Dr. Weir, the Rat’s Progress provided a powerful tool of epidemiological reasoning regarding the spread of plague between contiguous areas, through the agency of migrating rats. However, it would be mistaken to see the migratory rat as simply a figure that provided a solution to an epidemiological problem (that of contiguous propagation). For in the way in which it brought an animal, a disease, and space together, the figure of the migratory rat in fact marked a radical transformation in epidemiological reasoning.

## Shifting Kinetic Dramaturgies

The migratory rat was seen as involved in a dramaturgical act of survival, to extend an idea of Charles Rosenberg.^28^ Panicking in the face of the mass death brought about by epizootic plague, rats attempted to escape certain death by means of a mass exodus from an infected locality, which in turn spread the disease and death itself to new, invaded locations not only among their kin but also to grain, cloths, the soil, and humans. The figure of the migratory rat dramaturgically rhymed with, but also differed radically from, earlier configurations of the animal in relation to plague. In particular, its focus on rats responding to plague by means of an altered mobility rhymed with the image of the staggering rat, which, arising in missionary approaches to plague in 1870s Southwest China, had become a standard trope in medical works on the disease by the 1890s.^29^ In the words of James Cantlie, a leading plague expert at the time, “Before, or it may be during, an epidemic of plague, or before the individuals in any particular house in an infected locality are stricken, the rats leave their haunts and seek the interior of the house. They seem careless of the presence of man and run about in a dazed way with a peculiar limping jerk or spasm of their hind legs.”^30^

**Figure 1.**
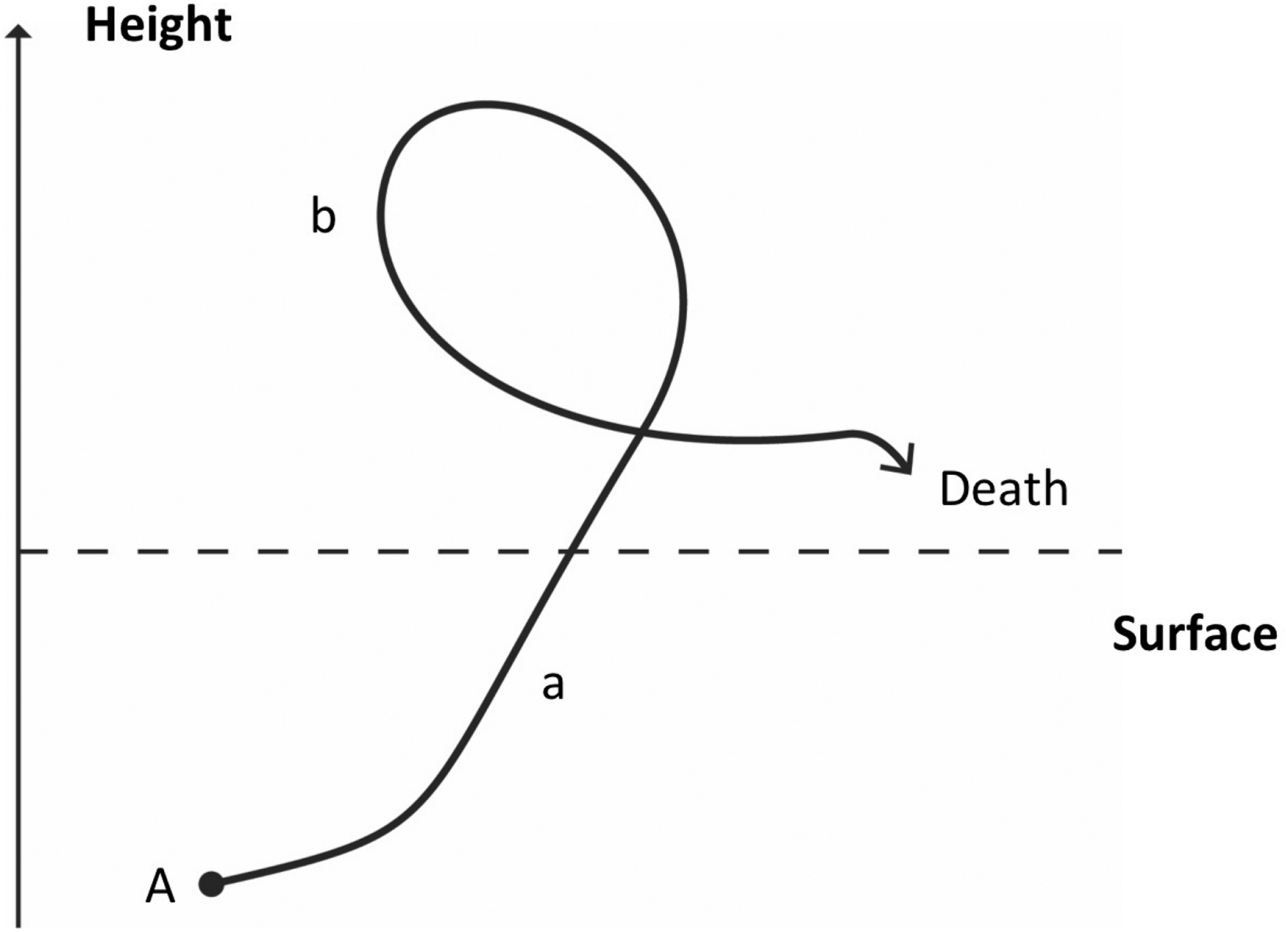
The movement of the staggering rat; design by the author.

Shared by both the figure of the staggering rat and the figure of the migratory rat was a kinetic dramaturgy, which instituted rats into animals that are ontologically conditioned by plague: in other words, as animals whose very nature is defined by the disease they carry. In the case of the staggering rat, the kinetic dramaturgy ontologically tying rats and plague together involved two movements (Fig. 1). First was an underground-overground/inside-out movement of plague-infected rats (a in Fig.1), as these were supposed to abandon their subterranean, out-of-sight abodes (A in Fig.1) and to come onto the surface of built structures, into the light as it were, becoming visible to humans. Second was a pirouette movement (b in Fig.1), whereby rats swirled around themselves and then fell down dead. This dramatized double movement, which authors at the time maintained was the direct impact of plague on rats, involved rats undergoing an ontological transformation where upon they abandoned their natural character as elusive, secretive beings so as to voluntarily reveal themselves to their enemies (humans) and, having thus betrayed their nature, perform a danse macabre and perish.

**Figure 2.**
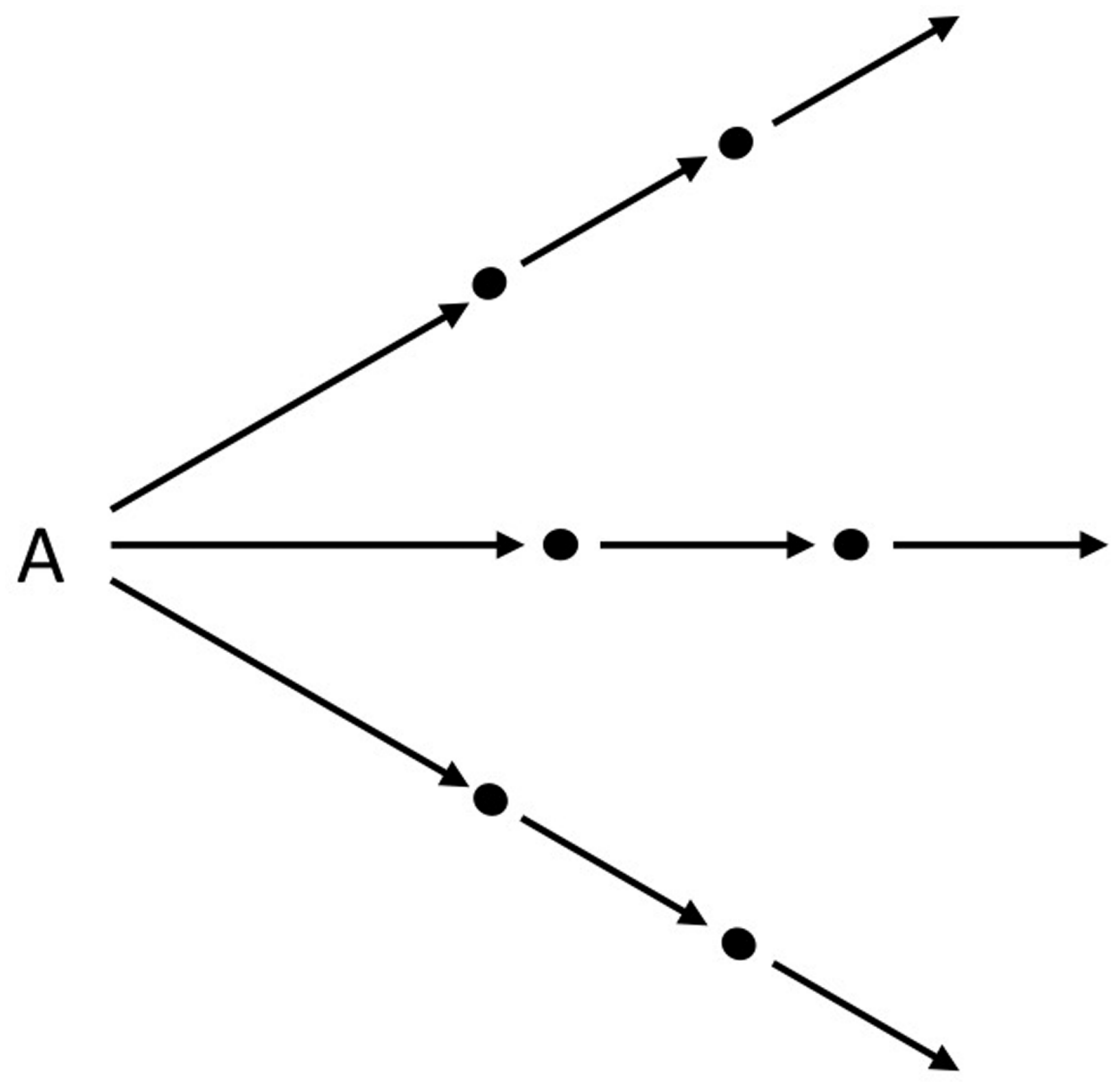
The movement of the migratory rat; design by the author.

In the case of the migratory rat, the kinetic dramaturgy was much simpler. Here the movement induced by plague was a linear one, forcing rats to abandon plague-infected localities not vertically (from underground to overground) but horizontally (whether underground or overground) so as to reach hitherto uninfected localities. This was an exodus from the location of origin and an invasion of destination locations, which were in turn abandoned once infected, leading to what in the scheme of the Rat’s Progress was described as both a simultaneous infection of different localities (in so far as not all rats would escape in a single direction) and a sequential infection of adjacent localities (Fig.2).

The movements of the two rat-figures unfold in and configure different spaces. The figure of the staggering rat moves in a three-dimensional, usually domestic space. On the one hand, this is a self-enclosed and self-referential space: a proper stage with no continuity or connection to other spaces. On the other hand, it is a space that is defined by a seen/unseen dichotomy that corresponds to a hidden, subterranean space of animals/vermin and a visible, overground space of humans. The movement of rats from their designated realm to the realm of humans thus marks an ontological invasion, which in structural terms involves the introduction of what Mary Douglas called “matter out of place.”^31^ At the same time, this strange movement may also be seen as a reiteration of long-standing narratives of animal deaths in the context of plague functioning as what Christian Jouhaud, Dinah Ribard, and Nicolas Schapira have identified as spectacles/indices of divine bane or scourge (*fléau*), being articulated as it was in French Catholic missionary narratives.^32^ It is, in other words, a movement that represents and produces not infection but pollution and scourge, or indeed infection as a by-product of pollution/scourge insofar as the epidemiological reasoning involved was underscored by ideas about miasma. The kinetic dramaturgy of the staggering rat may thus be said to, on the one hand, reveal the existence of plague, but also to maintain the animal/human divisions that were fundamental to both the missionary theology within which this figure emerged, and Victorian sensibilities in relation to which the figure would be further developed in the context of 1870s and 1880s medical literature.

By contrast, the figure of the migratory rat moves primarily within a two-dimensional, urban space. This is a space that is no longer closed upon itself, but open-ended and fundamentally inter-connected. It is a space that no longer reflects seen/unseen or animal/human dichotomies. By moving in it, rats do not reveal anything. Rather they carry something with them – plague – and spread it to ever newer and more distanced locations, transforming them in turn into infected ones by means of providing a catalytic connector among various pestigenic agencies (soil, humans, grain, etc.) contained therein.

At the same time, the two kinetic dramaturgies involved radically different temporalities. In the case of the staggering rat, this was one of revelation and catastrophe. The rat first revealed itself and plague to humans (upward/outward movement), and then acted as a metonymy of finitude or death by gyrating upon itself and dying in the open. In other words, it performed a veritable catastrophe, in the Greek meaning of the term, as a movement of one turning and collapsing upon one’s person. True to its missionary origins, the temporality here is clearly apocalyptic: it was an end-time oriented temporality, originally meant to rhyme with other catastrophes (war, famine, cannibalism) in Yunnan so as to provide a religious basis for colonization and conversion.^33^ In its later, medicalized versions (e.g., Cantlie quoted above), the out of place and out of character double movement of the staggering rat was seen as an epidemiologically favorable invasion of human space insofar as it revealed the hitherto invisible workings of plague in a given locality – the staggering rat being thus transformed from a portent of end-time to a sentinel of an imminent plague outbreak. By contrast, in the case of the migratory rat the temporality involved was one of perpetuity – the linear movement of the rat in space being also a linear movement of plague in time – with no end in sight. Thus, whereas the staggering rat was a figure that was always already metaphysical, insofar as it revealed the secret workings of God or Nature, the migratory rat marked a secularization of epidemic temporality in late nineteenth-century pandemic imaginaries as a time that followed the proverbial arrow of progress, albeit in this case Rat’s Progress. The movement of the migratory rat revealed no secrets; it was not a signifier of a higher-order or out-of-sight phenomenon, will or process. Rather it was in and of itself an epidemiological mechanism of the propagation of plague across space and time, marking for the first time the historical as well as epidemiological agency of rats as epidemic villains.

What made the two kinetic dramaturgies radically different to one another may thus be said to be the way in which they configured invasion. Both movements were invasive insofar as they involved the introduction of an animal or of a number of animals where they did not exist before. And both invasions were believed to be ontologically conditioned, resulting from the impact of plague on the nature of rats. However, by contrast to the invasion effected by the staggering rat, which brought about an ontological effect (pollution/scourge), i.e., *an effect of the order of its cause*, the impact of the invasion effected by the migratory rat was not ontological but rather epidemiological (infection) and thus not analogous to its cause. The move from the staggering to the migratory rat thus marked a radical transformation in approaching the relation between rats and plague, fostering a secularized and medicalized epidemiological reasoning and imagination.

There is no indication that medical and public health actants on the ground in British India reflected on these complex dramaturgical and temporal affinities and incongruities between the two rat figures. However, they did note that the figure of the staggering rat provided a logical obstacle to the acceptance of rat migration as a result of plague, or as a cause of its spread. One of the most vocal proponents of the migratory rat theory was Dr. C.H. Freeman Underwood, who on 18 February 18 1899 testified to the Indian Plague Commission that he had heard about an “army of rats walking from place to place” and a migration of rats from one to other side of a street in Mandvi, Bombay.^34^ Being the first out of two such commissions to be formed in India during the third plague pandemic, the investigations of this commission involved over seventy sittings undertaken between November 1898 and March 1899 across India during which 260 witnesses were questioned.^35^ Key to these exchanges was the definition of witnessing as excluding generalizations or anything not directly observed by the respondents.^36^ Indeed, Nicholas Evans has shown how witnesses to the Indian Plague Commission were unequivocally brought back to the order of observed facts when they tended to “wander” towards generalities and theories.^37^ Equally the interview panel often challenged witnesses to give more detailed, evidence-based accounts of their observations, which in some cases resulted in revelations of the paucity of the latter, or of witnesses having embroidered their initial accounts with speculations or hearsay. As Evans argues, “The truth that the commissioners sought thus emerged through a particular form of speech, and could not be gleaned from prior studies, pre-submitted précis, or published papers.”^38^ It is in this dialogical and epistemological context that we need to examine Freeman Underwood’s evidence to the Indian Plague Commission in relation to the migratory rat. Questioned about how “the exodus of rats” during the epidemic was ascertained, Freeman Underwood stated that having heard of their migration, he “went to verify the fact” and indeed saw rats “travelling over the causeways.”^39^ Appearing doubtful of this observation, the Commission asked him again, “Have you seen travelling along the streets?” “Yes,” responded the physician, “I have seen them running along the causeways.”^40^ However, the Commission seemed to be finding it hard to believe this story on account of the well-established staggering rat trope: “But is it not the fact that when rats are dying of plague they, in a dazed condition, leave their holes, so that more would be seen than in ordinary times?”^41^ Taken aback, Freeman Underwood retorted by overcompensating: “I was the first that pointed that out. They were in a state of plague intoxication and they did not care where they went; and they did not seem to notice human beings if they came near them or even kicked them.”^42^ The confrontation between the Commission and Freeman Underwood soon exposed the latter’s evidentiary basis to be feeble. Under pressure by an increasingly aggressive interviewer, he admitted that he had not actually gone to the causeway with the purpose of seeing the rats, but was simply passing by. Asked how many rats he observed migrating and to what direction, he made a few contradictory attempts at answering before the exasperated interviewer, having lost all interest in what now looked like an account that brought together hearsay and doubtful observations, ended the interview. Yet, besides being an excellent example of the dialogical and agonistic production of evidence in the context of the Indian Plague Commission, the interview points at a key if rarely directly articulated epidemiological aporia: “Does the fact of a rat being seen where it never was seen before show that it comes from another place?”^43^

## Mapping Rats

**Figure 3.**
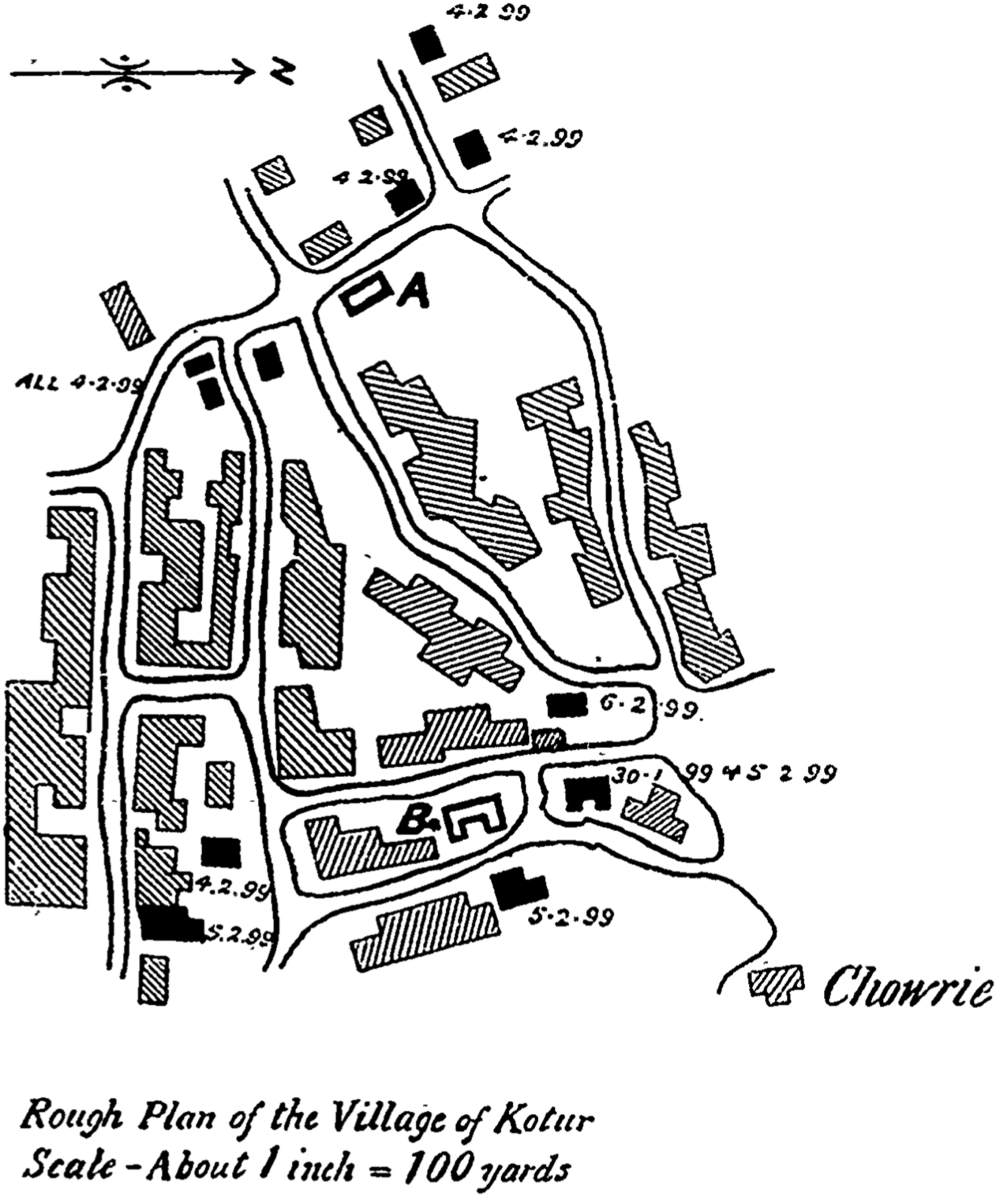
Colomb’s Kotur map; HCPP, Cd.141; Image published with permission of ProQuest LLC. Further reproduction is prohibited without permission.

A key method used to answer this question and demonstrate rat mobility over land and its epidemic impact was maps, marking some of the first steps in employing cartography to discuss plague and its epidemiology during the third pandemic. Captain A.F.C. Colomb of the Indian Staff Corps, interviewed by the Indian Plague Commission on March 1899, provided an account of a plague outbreak in Kotur village, in today’s Karnataka, at the end of January of the same year.^44^ He marked a particularity of the outbreak being that all deaths in the western side of the village, inhabited by “low caste families,” took place on the same day (4 February), while most deaths in the Eastern side, inhabited by “high caste families,” took place in the course of three consecutive dates (4-6 February). Colomb used a map (Fig. 3) showing houses where human deaths occurred (filled in black) and houses where ratfalls were observed (outlined in black).

On the map, the two houses where ratfalls took place, one on the west and the other on the east side of the village, were marked A and B respectively. Colomb admitted to not knowing the date of the ratfall in house A but asserted that one took place in house B on 4 February. As the groups living in the two quarters were said to have no contact with one another, it was concluded that, “rats were the agency for the spread of infection.”^45^ As regards the western part of the village, it was argued that rats imported plague to house A, where however no human cases occurred, supposedly due to it being immediately evacuated. Nonetheless, Colomb reasoned that the simultaneous death in that part of the village indicated that, “the infection had been evenly diffused and evidently carried by rats.”^46^ It was caste that led Colomb to conclude that rats also spread plague between the western and eastern quarters of the village.^47^ Questioned repeatedly about this point by the Commission, he argued that the shepherds who composed the population of the western quarter could have no contact with the high-caste population of the eastern quarter. He then procured another map to demonstrate a case where human agency was to blame for the propagation of plague. This was a plan of the plague camp at Kotbagi, which he argued was free of rats. The map showed a timeline of infection, starting on 21 January, which did not follow a linear spatial pattern, something attributed to the disease being spread by women visiting plague patients in so-called infected houses.

Epidemic cartography was at the time on the rise in India in response to the plague epidemic, with a particularly systematic application in the case of Bombay.^48^ Having examined the colonial cartography of plague in Bombay between 1896 and 1914, Evans has argued that disease maps, on the one hand, operated within the confines of an epistemic desire for certainty, while, on the other hand, functioned as “records of doubt and indecision” regarding the epidemiology of the disease and the best ways to control it.^49^ Colomb’s are some of the first maps to be used comparatively in order to demonstrate two cases where the spread of plague was attributable to different agents. Both in the evidence of the Indian Plague Commission and in reports on plague by local commissions on the disease, such maps proved catalytic in establishing a spatially-oriented visual evidentiary apparatus about the propagation of plague.

**Figure 4.**
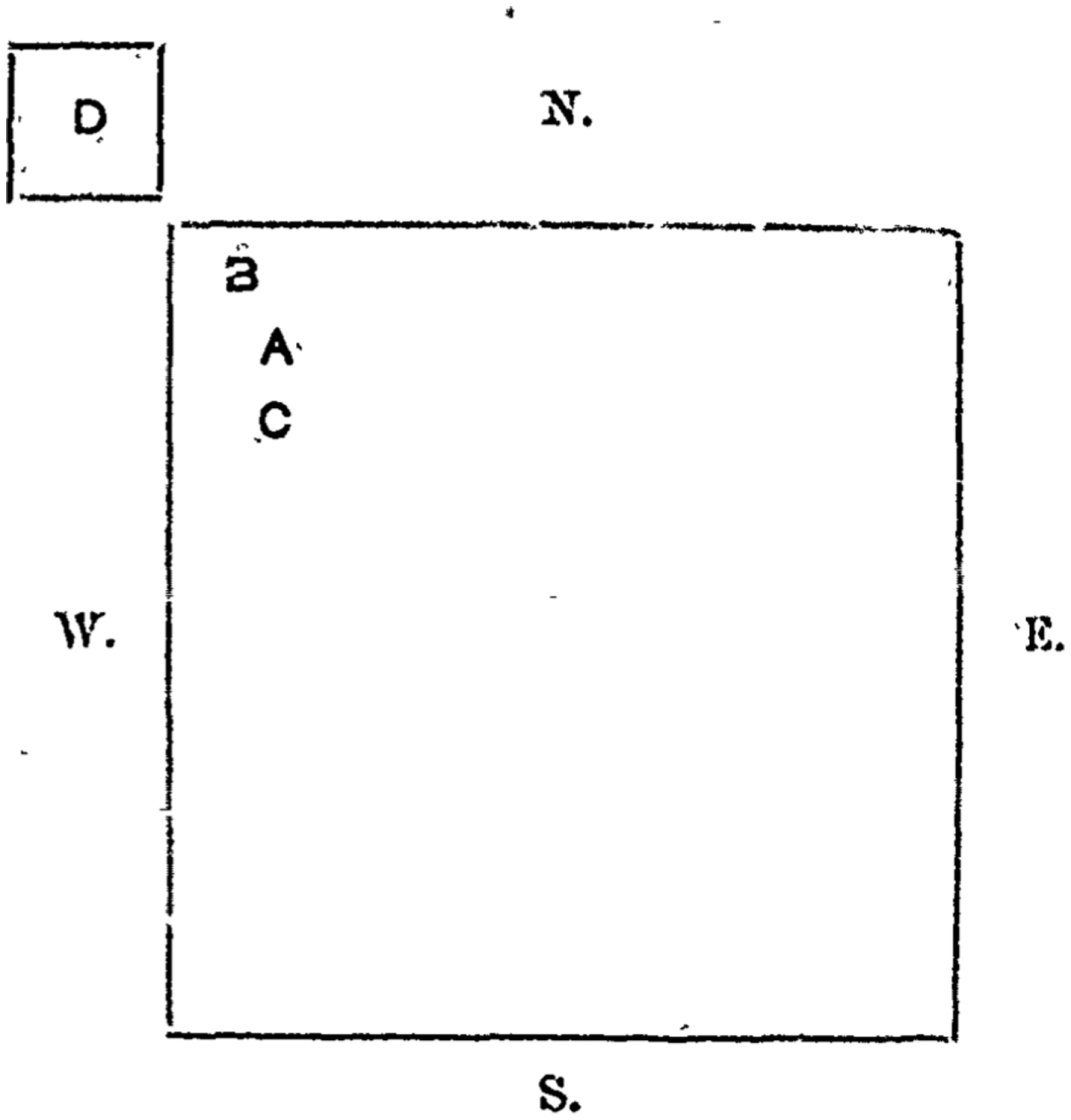
Dadachanji’s Bulsar map; HCPP, Cd.140; Image published with permission of ProQuest LLC. Further reproduction is prohibited without permission.

Yet rat cartography was not the monopoly of British doctors in India. In at least one case we have evidence of their use in order to contest British colonial framings of the disease, particularly as related to its spread between villages, cities or towns, and the role of rats in this process. Most colonial medical experts operating in British India at the time reserved the role of inter-city spread of plague not for rats but for humans, who were often seen as introducing plague in an entirely new location and therein able to infect both rats and humans, often through the mediating agent of grain.^50^ Indeed, the idea that it was humans who spread plague between distanced locations was instrumental in putting in place and legitimizing some of the most intrusive and draconian epidemic control measures during the first years of the pandemic in India, including the removal of the populations of entire towns and cities into quarantine camps.^51^ Khan Bahadur P.H. Dadachanji, Assistant Surgeon at Bulsar, sought to contest this colonial medical consensus, asserting that, “the village of Bhagda Khurd, which is about three-quarters of a mile distant from Bulsar, affords an instance of a town infecting the villages in its neighbourhood through migrating rats.”^52^ Testifying to the Indian Plague Commission, the doctor had trouble convincing it of this: “You say it spreads from the town to a village in the neighbourhood by the migration of infected rats, and subsequently that the rats migrate in that direction, because the disease went in that direction. I cannot see the point of your argument,” pressed the interviewer, perhaps not quite expecting a detailed response, which is exactly what was forthcoming.^53^ For Dadachanji produced a spatial diagram (Fig. 4) with which he proceeded to explain his epidemiological reasoning:

Supposing this square represent the town of Bulsar. The north-west end of town was infected first of all. The first infected street – Dheberwad - was at A, and the second street, Taiwad, at B, the street on the other side of Dheberwad, called Ghanchiwad, at C, and the village of Bhagda at D. Now the infection spread from A to B., and not to C, though the latter was also quite close; and before it attacked C or the other parts of the town rats began to die in the village of Bhagda. As, shortly after the appearance of the disease in Dheberwad (A) rats began to die in Taiwad (B), and then in Bhagda (D), situated in the same direction, and as the disease, as far as could be made out, could not have gone either to B or to D in any other way, the inference is that it was taken there by rats.^54^

While the Commission was not eventually convinced by the notion that rats were spreading the disease in this manner, Dadachanji’s testimony represents an ingenious moment where an Indian physician employed cartography in order to achieve a “conjunction of analytic presentation and experimental argumentation in a visual exposition” that contradicted the outbreak narrative of the colonial masters.^55^ This is an excellent example of what Lukas Engelmann has called a “spatial diagram,” which in the context of epidemiological reasoning “is perhaps best understood to be a hybrid between maps and functional diagrams,” operating at the “conjunction of analytic presentation and experimental presentation in visual exposition.”^56^ Indeed Dadachanji’s diagram may be said to be an experimental system, in Hansjörg Rheinberger’s sense of the term, which attempted to bring things together not so much as to showcase causality, but so “as to emphasize […] uncertainty, crisis, and invisibility.”^57^ Here, in the case of one of the first zoonotic diagrams in the history of epidemiology, we need to consider what I have elsewhere argued is “the relation between geometric and iconographic components (lines and figures) of the diagram and its surface as a meaningful one; indeed, as a relation whose apparent invisibility is an important analytical component of its articulation.”^58^ For what is decisively non-visible in a graphic manner in Dadachanji’s diagram is its protagonist: the rat. Whereas in Colomb’s map, rats are rendered visible through the contour coloring of houses where ratfalls were observed, here rats are not graphically described. And yet, by not being visibilized, in Dadachanji’s case rats may be said to remain always already *potential* in the geometric configuration of the diagram’s elements: a visual abstraction that reinforces the symbolic power of the rat as an animal that inhabits and occupies our world unseen, and at the same time as an animal with a unique propensity or indeed agency to connect and relate.

## Vagrant Rats

The debate over the rat’s cross-village migratory capacities had direct practical consequences, as summarized in Surgeon-Captain Smith’s note that, were plague to be carried by rats between villages, then all plague-control efforts would be futile, whereas if rats only spread plague within a given location, “then the disease is amenable to human control.”^59^ Hence it is not surprising that, rather than being limited to medical publications, the debate about the migratory rat soon spilled over into the daily press. I only have space here to discuss one case, which, being authored by a colonial celebrity bore particular weight in terms of disseminating the notion of the migratory rat beyond the scientific community. On 19 June 1899 the Bombay-based civil servant Edward Hamilton Aitken, aka Eha – best known at the time for his popular books *The Naturalist on the Prowl* (1894) and *Behind the Bungalow* (1889, with six editions by 1897) – published his reflections on the notion that rats could spread plague between villages. As Jason Sandhar has discussed in detail, Aitken was exemplary of Anglo-Indian civil servants turned naturalists in that he humoristically inflected the “nature essay” to both reinforce colonialist, racist stereotypes and hierarchies and to reflect colonial anxieties about political power and human/non-human mastery: “caricatures of India’s animals and people both lampoon and affirm the colonial class’ anxieties about a vast and unruly subcontinent that the colonial government cannot entirely comprehend or control.”^60^ It is thus interesting that in the case of the rat, rather than investing on what following Jonathan Saha we could call the “willfull behaviour” of the animal, Aitken’s article, appearing in *The Times of India*, directly contradicted Smith’s argument about rat intelligence:

If rats do take alarm and decamp when a few of their number have died by the plague, they are probably actuated not by reason or intelligence, but by those perceptions in respect of which man is generally far inferior to the lower animals. We do not attribute high class intellectual and reasoning capacity to ants because they know, when we do not, that it is going to rain, and remove their so-called “eggs” out of danger of inundation.^61^

Rather than seeking evidence on the anticolonial agency of rats, or reading them simply as a commentary on the vicissitudes of colonial power, we here need to take Aitken’s writings ethnographically seriously. Aitken, who in his popular *A Naturalist on the Prowl* had painted a nefarious picture of rats, raised an important point that seemed to be neglected by most scientific publications at the time: the differences between rat species.^62^ He argued that studies led by Oldfield Thomas had shown that “the common house rat of India” was “a variety of *Mus alexandrines*, the Egyptian rat,” itself being “variety of *Mus rattus*, the old black rat of England.”^63^ The point, according to Aitken, was crucial as while “The Norway rat lives more on the ground, thrives on sewer gas, and is decidedly more carnivorous than the other. The Black rat, as Buckland tells us, ‘delights not in low haunts, such as cellars and pigsties, nor does he burrow and run into holes, &c., but lives chiefly in the ceilings and wainscots of houses, and under the ridge-tiles and behind the rafters of outhouses.’”^64^

This was the first time during the third plague pandemic that a discussion of the ethology of different rat species was being discussed in relation to the question of the rat’s relation to plague. According to Aitken, the Indian house rat was an amble acrobat who preferred living in trees, coming into houses during the monsoon to avoid the rain: “When the monsoon is over they go out again and lead a vagrant life, feeding on seeds and fruits and birds’ eggs, and even on birds which they catch sleeping. They climb palm trees and do so much damage to young cocoanuts that in Canara, where much attention is given to cocoanut cultivation, every tree is protected by a belt of thorns round the trunk, eight or ten feet from the ground.”^65^ Confronting Smith’s idea of rat colonies, Aitken’s pen assumed the well known, acerbic qualities that made him such a popular author: “There is no such thing as a colony of rats. A colony of tigers would be as likely. Until they are full-grown the members of one family may keep together, but every full-grown rat is an Ishmael. Where food is plentiful they will congregate in large numbers, but when not feeding they will be fighting.”^66^ So “vagrant” a life did such rats lead, that according to Aitken, “Any individual among them, catching the infection of plague at one end of the village, may die at the other end, after visiting every cranny of half the houses between.”^67^ In writings such as Aitken’s, rats were configured as a sagacious species for which invasiveness assumed new ontological qualities that both complemented and differed from already established ones. Rather than simply being situationally invasive – in the sense of moving or being moved, under a specific timeframe, into environments where they do not originate or belong – rats were, in other words, seen as being in and of themselves invasive. Aitken stressed precisely the transgressive and connecting propensity of the animals, the being-for-invasiveness of rats, in asserting their propensity to infect the floors, clothes bedding and food of houses they visit, “for rats pry into everything.”^68^ While still considered vital as regards the propagation of plague, invasiveness thus became an autonomous trait of rats as it was no longer seen as a result of plague unto their nature, but as an essential and constant part of the latter - one may be tempted to say, of their species-being.

## Conclusion

If during the first years of the third plague pandemic in British India, the rat was a charismatic player in, on the one hand, inter-relating supposedly pestigenic agents, and on the other hand, inter-connecting plague-infected and plague-free locations, this was to a great extent because it functioned as a material, organic topos that, as Evans has argued, allowed colonial governments to integrate bacteriological and sanitary approaches to plague, blame Indian lifeways for the propagation of the disease, and “re-establish the fixed racial and caste categories through which they ruled.”^69^ Yet, at the same time, as I have shown in this article, the figure of the migratory rat made possible an array of other epistemic and political processes and practices. First, the migratory rat embodied and in turn fostered a new, secularized idea of epidemic disease, as a process in and for itself, which unfolded in a linear, future-oriented temporality. Nothing embodied this new idea of epidemics better than the Rat’s Progress, whereupon rats as vectors of plague over an endless chronotope replaced rats as emblems of epidemics as events marking an apocalyptic end-time (as had been embodied in the figure of the staggering rat). Second, questions around the migratory rat fostered practices of epidemiological reasoning that necessitated engaging in complex syntheses and negotiations of evidence, including emerging practices of disease cartography. Third, these practices were not the monopoly of colonial doctors; instead, Indigenous medical experts also took recourse to them, in some cases in order to contest colonial epidemiological consensus about the means of the propagation of plague. Finally, as discussions about the migratory rat and its role in the spread of plague unfolded both in the lay and in the medical press, the migratory rat became invested with attributes of invasiveness that assumed ontological qualities.

In the years following the period examined in this article, with plague becoming truly pandemic, medical and political interest shifted attention away from its land-based propagation, and transformed the migratory rat into a maritime figure. This required significant symbolic, epistemic, and political reinvestments of rats as epidemic agents and villains. Invasiveness, when it came to rats’ relation to plague, would by 1905 become a trope reworked with xenophobic images and invested with questions of sovereignty and geographic blame. Far more than simply reproducing pre-bacteriological configurations of the rat as vermin, it was on the epistemic, ideological, and ontological grounds set in place through the emergence of the migratory rat as a land-based figure in British India that this new, global framing of seaborne, invasive, pestigenic rats would have to rely.

## Footnotes

1 Myron J. Echenberg, *Plague Ports: The Global Urban Impact of Bubonic Plague, 1894-1901* (New York: New York University Press, 2007); Christos Lynteris, *Visual Plague: The Emergence of Epidemic Photography* (Cambridge MA: MIT Press, 2022), see chapter 4.

2 Ibid.

3 See for example, Katharine Dean, Fabienne Krauer, and Boris V. Schmid, “Human Ectoparasites and the Spread of Plague in Europe during the Second Pandemic,” *PNAS* 115 (2018): 1304-1309.

4 Lynteris, *Visual Plague*.

5 Lukas Engelmann and Christos Lynteris, *Sulphuric Utopias: A History of Maritime Fumigation* (Cambridge MA: MIT Press, 2020); Jules Skotnes-Brown, “Scurrying Seafarers: Shipboard Rats, Plague, and the Land/Sea Border,” *Journal of Global History* 18 (2023): 108-130.

6 Lynteris, *Visual Plague;* Engelmann and Lynteris, *Sulphuric Utopias*.

7 Christos Lynteris, “The Figure of the Staggering Rat: Reading Colonial Outbreak Narratives Against the Grain of ‘Virus Hunting,’” *Journal of the History of Medicine and Allied Sciences* (2024): https://doi.org/10.1093/jhmas/jrae004.

8 David Arnold, *Colonizing the Body: State Medicine and Epidemic Disease in Nineteenth-Century India* (Cambridge: Cambridge University Press, 1993); Sanjoy Bhattacharya, “Plague in Bombay: Responses to Colonial Authority Control Measures,” *Wellcome History* 35 (2007): 1-24; Ian Catanach, “Poona Politicians and the Plague,” *South Asia: Journal of South Asian Studies* 7 (1984): 1-18; Ian Catanach, “’The Gendered Terrain of Disaster’? India and the Plague, c. 1896–1918,” *South Asia: Journal of South Asian Studies* 30 (2007): 241-267; R. Chandavarkar, “Plague Panic and Epidemic Politics in India, 1896–1914,” in *Epidemics and Ideas: Essays on the Historical Perception of Pestilence*, ed. Terence Ranger and Paul Slack (Cambridge: Cambridge University Press, 1992), 203–240; Samuel K. Cohn, *Epidemics: Hate and Compassion from the Plague of Athens to AIDS* (Oxford: Oxford University Press, 2018), esp. chapters 14, 15, and 16; Ira Klein, “Plague, Policy and Popular Unrest in British India,” *Modern Asian Studies* 22 (1988): 723–755; Projit Bihari Mukharji, *Nationalizing the Body: The Medical Market, Print and Daktari Medicine* (London: Anthem Press, 2009), esp. chapter IV; Aditya Sarkar, “The Tie That Snapped: Bubonic Plague and Mill Labour in Bombay, 1896–1898,” *International Review of Social History* 59 (2014): 181-214; Natasha Sarkar, “Plague in Bombay: Response of Britain’s Indian Subjects to Colonial Intervention,” *Proceedings of the Indian History Congress* 62 (2001): 442-449.

9 Frederique Audoin-Rouzeau, *Les chemins de la peste: le rat, la puce et l’homme* (Rennes: Presses Universitaires de Rennes, 2003), esp. chapter 2, 3, and 9; Pratik Chakrabarti, “Commentary: An Experimental Theatre for Vaccines — Bombay in the Time of Plague,” *International Journal of Epidemiology* 42 (2013): 656–658; Sonali Dhanpal, “The Making of a Model Town: Planning in a Princely City and the All-India Sanitary Conferences,” *Planning Perspectives* 38 (2023): 467-497; Christos Lynteris, “Pestis Minor: The History of a Contested Plague Pathology,” *Bulletin of the History of Medicine* 93 (2019): 55-81; Projit Bihari Mukharji, “Cat and Mouse: Animal Technologies, Trans-imperial Networks and Public Health from Below, British India, c. 1907–1918,” *Social History of Medicine* 31 (2017): 510–532; Matheus Alves Duarte da Silva, “From Bombay to Rio de Janeiro: The Circulation of Knowledge and the Establishment of the Manguinhos Laboratory, 1894-1902,” *História, Ciências, Saúde- Manguinhos* 26 (2018): 375-378.

10 Prashant Kidambi, “‘An Infection of Locality’: Plague, Pythogenesis and the Poor in Bombay, c. 1896–1905,” *Urban History* 31 (2004): 249–267; David S. Barnes*, The Great Stink of Paris and the Nineteenth-Century Struggle Against Filth and Germs* (Baltimore: Johns Hopkins University Press, 2006), 3.

11 Mary Sutphen, “Not What, but Where: Bubonic Plague and the Reception of Germ Theories in Hong Kong and Calcutta,” *Journal of the History of Medicine and Allied Sciences* 52 (1997): 81–113; Colin McFarlane, “Governing the Contaminated City: Infrastructure and Sanitation in Colonial and Post-Colonial Bombay,” *International Journal of Urban and Regional Research* 32 (2008): 415–435.

12 Christos Lynteris, “A ‘Suitable Soil’: Plague’s Urban Breeding Grounds at the Dawn of the Third Pandemic,” *Medical History* 61 (2017): 343–357.

13 Rats had been first suspected as associated with plague in India in the first half of the nineteenth century, but not seen as a source of plague in humans. Christos Lynteris, “Mahamari Plague: Rats, Colonial Medicine and Indigenous Knowledge in Kumaon and Garhwal, India,” *Medical Anthropology* 41 (2022): 373-386.

14 Christos Lynteris, “In Search of Lost Fleas: Reconsidering Paul-Louis Simond’s Contribution to the Study of the Propagation of Plague,” *Medical History* 66 (2022): 242–263.

15 Ibid., 246.

16 On rats as vermin see, Lucinda Cole, “Plagues, Poisons, and Dead Rats: A Multispecies History,” in *The Palgrave Handbook of Animals and Literature*, ed. Susan McHugh, Robert McKay, and John Miller (London: Palgrave Macmillan, 2020), 589-603; Mary Fissell, “Imagining Vermin in Early Modern England,” *History Workshop Journal* 47 (1999): 1-29; Neil Pemberton “The Rat-Catcher’s Prank: Interspecies Cunningness and Scavenging in Henry Mayhew’s London,” *Journal of Victorian Culture* 19 (2014): 520–535; Kaori Nagai, “Vermin Writing,” *Journal for Maritime Research* 22 (2020): 59-74.

17 Wellcome Library, XWC, Report of the Bombay Plague Committee, appointed by government resolution No. 1204/720P, on the plague in Bombay, for the period extending from the 1st July 1897 to the 30th April 1898 (Bombay: Times of India, 1898), 204.

18 Peter Simon Pallas, *Novae species Quadrupedum e Glirium Ordine: Cum Illustrationibus Variis Complurium Ex Hoc Ordine Animalium*, Vol. 1 (Walther: Erlangen, 1778).

19 The notion would be systematically introduced in plague literature by Emil Zuschlag, *Le Rat migratoire et sa destruction rationnelle*, trans. M. Pierre Oesterby (Copenhagen: Impr. F. Bagge, 1903).

20 Nicholas H.A. Evans, “Blaming the Rat? Accounting for Plague in Colonial Indian Medicine,” *Medicine, Anthropology, Theory* 5 (2018): 15-42, 16.

21 M.E. Couchman, *Account of Plague Administration in the Bombay Presidency from September 1896 till May 1897* (Bombay: Government Central Press, 1897), 21.

22 Ibid., 21.

23 Paul-Louis Simond, “La propagation de la peste,” *Annales de l’Institut Pasteur* 12 (1898): 625-687, 656. For further discussion see Lynteris, “In Search of Lost Fleas.”

24 “General Plague Report No. 5017 of 1897, from A. C. Logan to F. S. P. Lely, Thána, 12, July 1897,” in Couchman, *Account of Plague Administration in the Bombay Presidency*.

25 “Notes by Surgeon-Major A.V. Anderson, Deputy Sanitary Commissioner, Western Registration District,” in Couchman, *Account of Plague Administration in the Bombay Presidency*.

26 Ibid., 2.

27 “General Plague Report No. 5017 of 1897, from A.C. Logan to F.S.P. Lely,” 9, 7.

28 Charles E. Rosenberg, “What Is an Epidemic? AIDS in Historical Perspective,” *Daedalus* 118 (1989): 1-17.

29 Lynteris, “Figure of the Staggering Rat.”

30 James Cantlie, “A Lecture on the Spread of Plague before the Epidemiological Society on December 18th 1896,” *Lancet* 149: 3828 (1897): 85-91, 85.

31 Mary Douglas, *Purity and Danger: An Analysis of the Concepts of Pollution and Taboo* (London: Routledge, 1993 [1966]).

32 Christian Jouhaud, Dinah Ribard, and Nicolas Schapira, *Histoire, littérature, témoignage. Écrire les malheurs du temps* (Paris: Gallimard, 2009).

33 Lynteris, “Figure of the Staggering Rat.”

34 House of Commons Parliamentary Papers (HCPP), Cd.141, Indian Plague Commission, 1898-99. Minutes of evidence taken by the Indian Plague Commission with appendices. Vol. III. Evidence taken from 11 February 1898 to 20 May 1899, 83.

35 For a discussion of the two commissions see Evans, “Blaming the Rat?”

36 Ibid.

37 Ibid.

38 Ibid., 22. On this trial-like production of evidence by the Indian Plague Commission see also, Jacob Steere-Williams, “Carbolic Colonialism: Plague, Public Health, and Disability in British India,” *Osiris* 39 (2024): 225-243.

39 HCPP, Cd.141, 83.

40 Ibid.

41 Ibid.

42 Ibid.

43 Ibid.

44 Cd.141, Indian Plague Commission, 1898-99. Minutes of evidence taken by the Indian Plague Commission with appendices. Vol. III. Evidence taken from 11 February 1898 to 20 May 1899.

45 Ibid., 230.

46 Ibid.

47 For a discussion of caste in the third plague pandemic in India see, Antara Chatterjee, “Caste in Epidemics in India: A Historical and Literary Reading,” in *Pandemics and Epidemics in Cultural Representation*, ed. Sathyaraj Venkatesan, Antara Chatterjee, A. David Lewis, and Brian Callender (New York: Springer, 2022), 245–266.

48 Nicholas Evans, “The Disease Map and the City: Desire and Imitation in the Bombay Plague, 1896-1914,” in *Plague and the City,* ed. Lukas Engelmann, John Henderson, and Christos Lynteris (London: Routledge, 2018), 116-138.

49 Ibid., 118.

50 See for example, “Notes on the Plague Operations in the Nawashahr Circle, Jullundur, July 1898. By Surgeon-Captain H. Smith, M.D., I.M.S., Plague Medical Officer, Nawashahr Circle,” in C.H. James, *Report on the Outbreak of Plague in the Jullundur and Hoshiarpur Districts of the Punjab, 1897-98* (Lahore: Civil & Medical Gazette Press, 1898), 161.

51 Aidan Forth, *Barbed-Wire Imperialism: Britain’s Empire of Camps, 1876-1903* (Berkeley: University of California Press, 2017).

52 HCPP, Cd.140, Indian Plague Commission, 1898-99. Minutes of evidence taken by the Indian Plague Commission with appendices. Vol. II. Evidence taken from 11January 1899 to 8 February 1899, 315.

53 Ibid.

54 Ibid., 316.

55 Tom Koch, *Disease Maps: Epidemics on the Ground* (Chicago: University of Chicago Press, 2011), 13.

56 Lukas Engelmann, “Configurations of Plague: Spatial Diagrams in Early Epidemiology,” *Social Analysis* 63 (2019): 89-109, 90, 93.

57 Ibid., 89; Hans-Jörg Rheinberger, “Experimental Systems: Historiality, Narration, and Deconstruction,” in *The Science Studies Reader*, ed. Mario Biagioli (New York: Routledge, 1999), 417–429.

58 Christos Lynteris, “Zoonotic Diagrams: Mastering and Unsettling Human-Animal Relations,” *Journal of the Royal Anthropological Institute* 23 (2017): 463-485.

59 “Notes on the Plague Operations in the Nawashahr Circle,” 161.

60 Jason Sandhar, “A Point that Escapes Darwin: Crises of Colonial Self in the Nature Essays of Edward Hamilton Aitken and Philip Robinson,” *Journal of Commonwealth Literature* 58 (2023): 692–706.

61 Anon [Eha]., “Rats and the Plague” *Times of India*, 19 July 1899, 4; Jonathan Saha, “Colonizing Elephants: Animal Agency, Undead Capital and Imperial Science in British Burma,” *BJHS Themes* 2 (2017): 169–89, 170.

62 Eha [E.H. Aitken], *A Naturalist on the Prowl or In the Jungle* (London: W. Thacker & Co, 1894).

63 Anon., “Rats and the Plague,” 4.

64 Ibid.

65 Ibid.

66 Ibid.

67 Ibid.

68 Ibid.

69 Evans, “Blaming the Rat?” 37.

